# Supplementary material for: Revisiting the role of oxidation in stable and high-performance lead-free perovskite-IGZO junction field-effect transistors
Source: Nat Commun. 2025 Aug 11;16:7427. doi: 10.1038/s41467-025-62770-2 (PMC12339744; doi:10.1038/s41467-025-62770-2)
Supplement: Supplementary file 1 — Supplementary Information [file 41467_2025_62770_MOESM1_ESM.pdf]

Supplementary Information for

Revisiting the role of oxidation in stable and high-performance lead-free perovskite-IGZO junction field-effect transistors

Seonkwon Kim[1], Su Hyun Kim[2], Hui Ung Hwang[3,4], Jeongmin Kim[5], Jeong Won Kim[3,4], In Cheol Kwak[1], Byeongjae Kang[6], Seungjae Lee[1], Sae Byeok Jo[7,8], Du Yeol Ryu[1], Hyunjung Kim[6], Jae-Min Myoung[9], Moon Sung Kang[10,11]*, Saeroonter Oh[12]*, Jeong Ho Cho[1]*

[1]Department of Chemical and Biomolecular Engineering; Yonsei University, Seoul, 03722, Republic of Korea.
[2]Department of Electrical and Computer Engineering, Sungkyunkwan University, Suwon, 16419, Republic of Korea.
[3]Korea Research Institute of Standards and Science (KRISS); Daejeon, 34113, Republic of Korea.
[4]University of Science and Technology (UST); Daejeon, 34113, Republic of Korea.
[5]Division of Nanotechnology, DGIST; Daegu, 42988, Republic of Korea.
[6]Center for Ultrafast Phase Transformation, Department of Physics, Sogang University, Seoul 04107, Republic of Korea.
[7]School of Chemical Engineering, Sungkyunkwan University (SKKU); Suwon, 16419, Republic of Korea.
[8]SKKU Institute of Energy Science and Technology (SIEST), Sungkyunkwan University; Suwon, 16419, Republic of Korea.
[9]Department of Materials Science and Engineering, Yonsei University; Seoul, 03722, Republic of Korea.
[10]Department of Chemical and Biomolecular Engineering, Sogang University; Seoul, 04107, Republic of Korea.
[11]Institute of Emergent Materials, Ricci Institute of Basic Science, Sogang University; Seoul, 04107, Republic of Korea.
[12]Department of Semiconductor Convergence Engineering, Sungkyunkwan University, Suwon, 16419, Republic of Korea.

*Corresponding authors: Moon Sung Kang (kangms@sogang.ac.kr), Saeroonter Oh (sroonter@skku.edu) and Jeong Ho Cho (jhcho94@yonsei.ac.kr)

Table-of-contents:

1. **Supplementary Fig. 1:** Fabrication process for the perovskite barriered junction field-effect transistor (b-JFET).
2. **Supplementary Fig. 2:** Optical microscopy image of the perovskite b-JFET.
3. **Supplementary Fig. 3:** Transfer characteristics of the perovskite JFET before the optimization process
4. **Supplementary Fig. 4:** Transfer characteristics of the perovskite IGZO-JFET measured at different drain voltages (V_{DS} = 1, 0.5, 0.1, 0.05 and 0.01 V).
5. **Supplementary Fig. 5:** Hall measurement of IGZO.
6. **Supplementary Fig. 6:** Frequency-dependent capacitance and phase angle measurements of perovskite devices.
7. **Supplementary Fig. 7:** Transfer characteristics of 100 perovskite b-JFETs.
8. **Supplementary Fig. 8:** Air stability measurements.
9. **Supplementary Fig. 9:** UV–visible spectra of the PEA_2SnI_4 films with different air exposure times.
10. **Supplementary Fig. 10:** Time-dependent photoluminescence measurements of perovskite films.
11. **Supplementary Fig. 11:** Gate bias stress stability results of the perovskite b-JFET device.
12. **Supplementary Fig. 12:** Cyclic stability results of the perovskite b-JFET device.
13. **Supplementary Fig. 13:** XPS depth profiles of perovskite films.
14. **Supplementary Fig. 14:** Ellipsometry measurements of perovskite films.
15. **Supplementary Fig. 15:** X-ray diffraction spectra of perovskite films.
16. **Supplementary Fig. 16:** GIXRD results of perovskite films.
17. **Supplementary Fig. 17:** EIS measurements of the perovskite b-JFETs.
18. **Supplementary Fig. 18:** Comparison of transfer characteristics with PMMA top gate dielectric.
19. **Supplementary Fig. 19:** Ultraviolet photoelectron spectra of the bulk PEA_2SnI_4 films after Ar sputtering for the etching of the surface layer.
20. **Supplementary Fig. 20:** Determination of Schottky barrier heights at Au-Pe0 and Au-Pe120 junctions through thermionic emission analysis.
21. **Supplementary Fig. 21:** Transfer curves of the Pe150-, Pe180-, and Pe210-based perovskite b-JFETs.
22. **Supplementary Fig. 22:** Transfer characteristics of perovskite b-JFETs as a function of annealing temperature.
23. **Supplementary Fig. 23:** Energy band diagrams and contour maps
24. **Supplementary Table 1:** Fitting parameters for ellipsometry measurements.
25. **Supplementary Table 2:** Benchmark comparison of IGZO FET-based inverters with solution-processed gate dielectrics.
26. **Supplementary References**

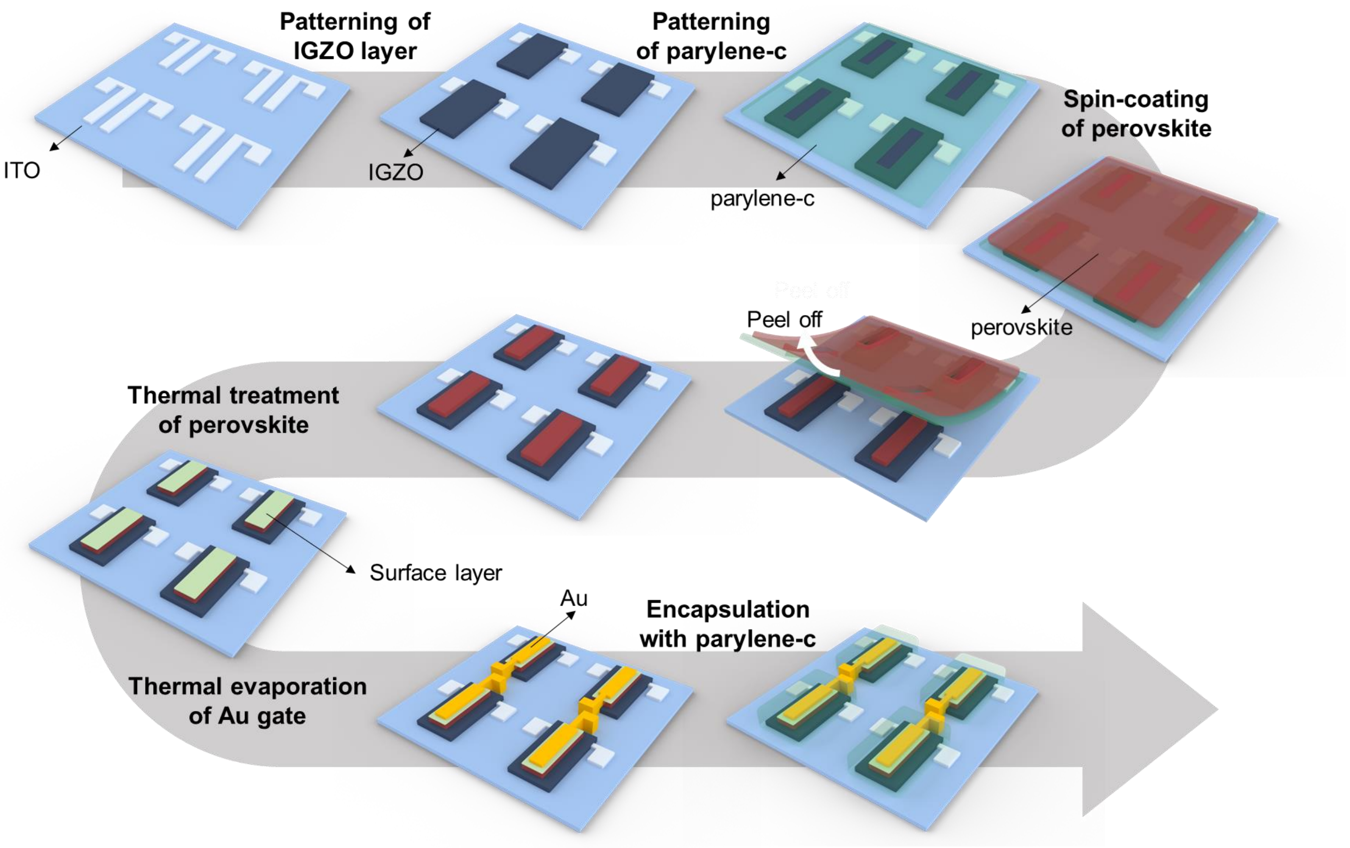

Supplementary Fig. 1. Fabrication process for the perovskite barriered junction field-effect transistor (b-JFET)

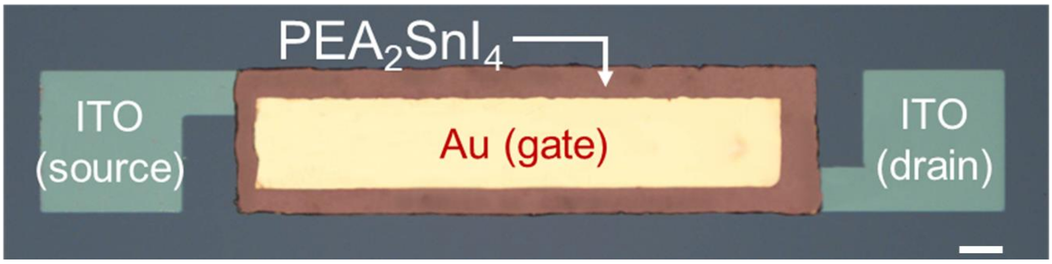

Supplementary Fig. 2. Optical microscopy image of the perovskite b-JFET
The scale bar corresponds to 100 μm.

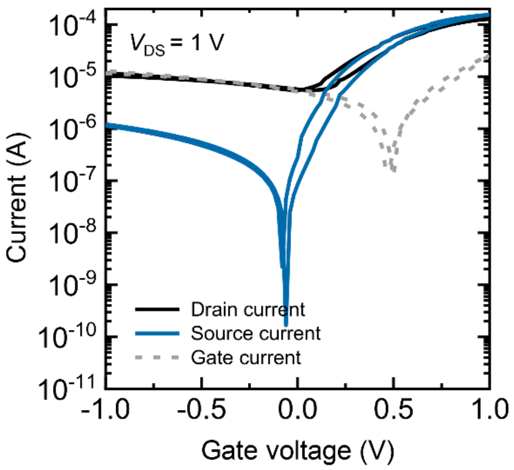

Supplementary Fig. 3. Transfer characteristics of the perovskite JFET before the optimization process

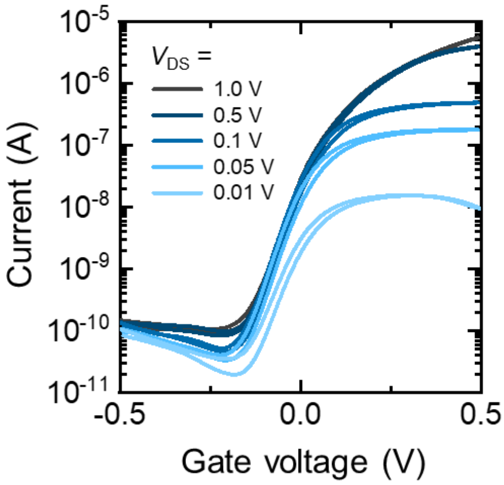

Supplementary Fig. 4. Transfer characteristics of the perovskite-bJFET measured at different drain voltages (V_{DS} = 1, 0.5, 0.1, 0.05 and 0.01 V)

At V_{DS} = 0.01 V, I_{DS} initially increases with V_{GS}, but shows a decline beyond ~0.4 V. This is due to gate-induced leakage current, which becomes comparable to the on-current at low drain bias. The gate leakage reaches the nA range at V_{GS} = 0.5 V, effectively reducing net drain current. This effect diminishes at higher V_{DS}, where the on-current dominates.

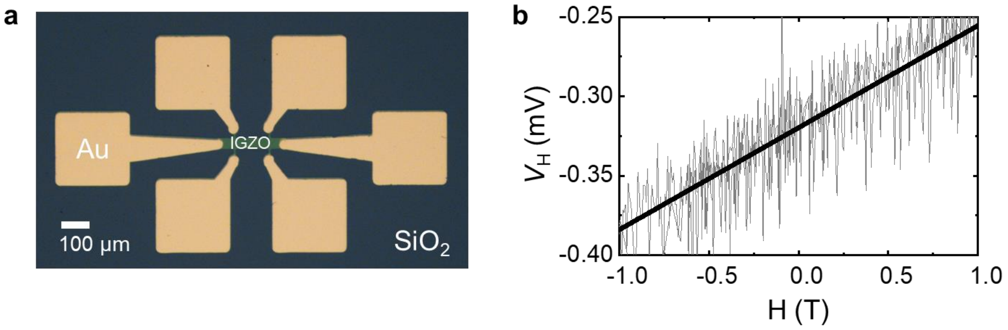

Supplementary Fig. 5. Hall measurement of IGZO

(a) Optical microscope image of IGZO hall bar device for estimating field-effect mobility. (b) Measured Hall voltage as a function of the external magnetic field H.

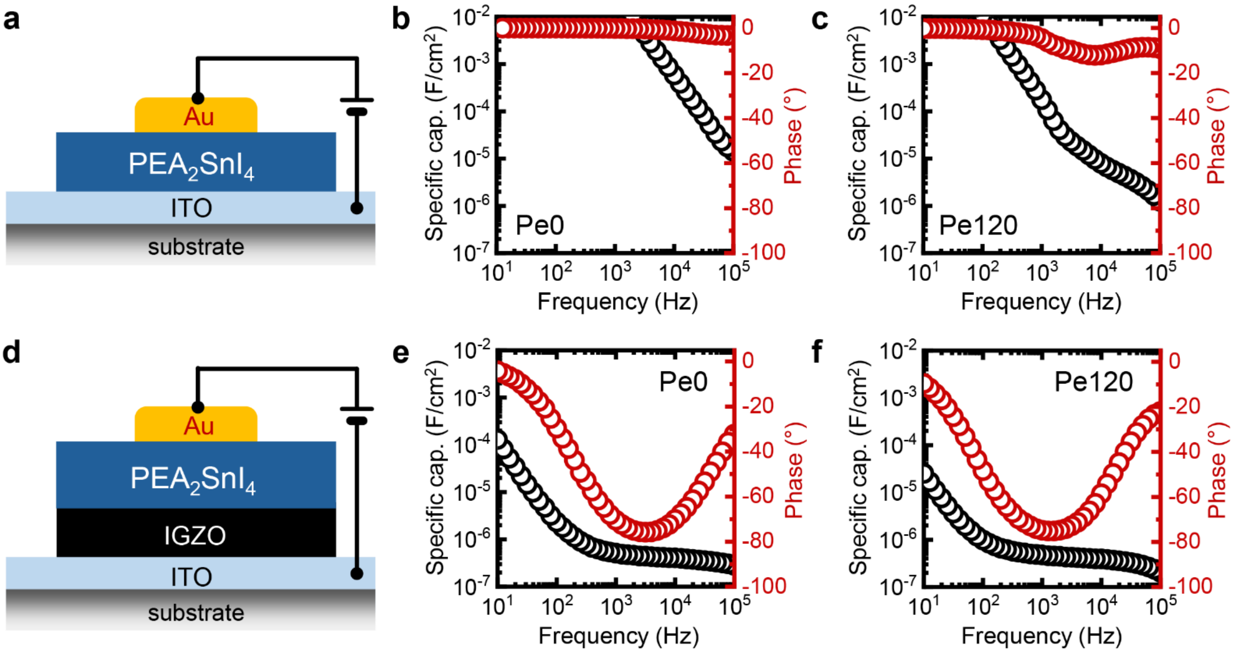

Supplementary Fig. 6. **Frequency-dependent capacitance and phase angle measurements of perovskite devices**

(a) Schematic of Au-perovskite-ITO device structure. (b), (c) Bode plots showing the capacitance and phase angle *versus* frequency for Pe0 and Pe120, respectively. (d) Schematic of Au-perovskite-IGZO-ITO device structure (e-f) Bode plots showing the capacitance and phase angle *versus* frequency for Pe0 and Pe120, respectively.

Analysis of the Bode plots demonstrates fundamental differences in device behavior with and without IGZO integration. The phase angle convergence to 0 degrees in devices without IGZO (**Supplementary Fig. 6a-c**) indicates a purely resistive characteristics. Conversely, IGZO-incorporated devices (**Supplementary Fig. 6d-f**) exhibit capacitive behavior with a non-zero phase angle, correlating with the semicircular response in the Nyquist plot (**Supplementary Fig. 17**). The observed semicircle in the intermediate frequency range (10-10^4 Hz) indicates charge accumulation at the perovskite-IGZO interface[8]. Based on these electrical characteristics, we classified our device as a JFET and accordingly employed a capacitance-independent mobility calculation method to ensure accurate mobility assessment.

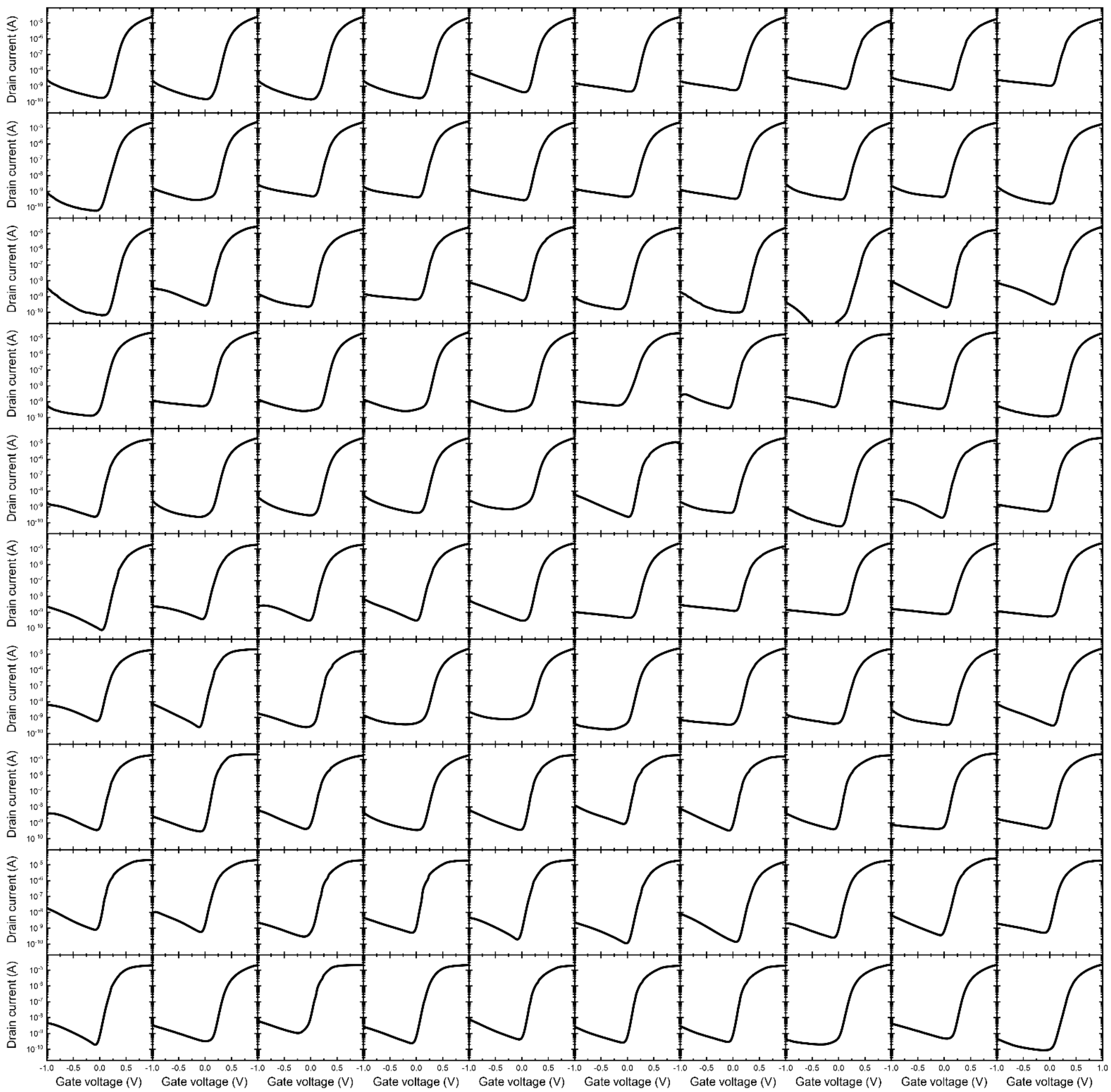

Supplementary Fig. 7. Transfer characteristics of a hundred perovskite b-JFETs

The channel length, width, and V_D of all the devices were 100 μm, 1000 μm, and 1 V, respectively.

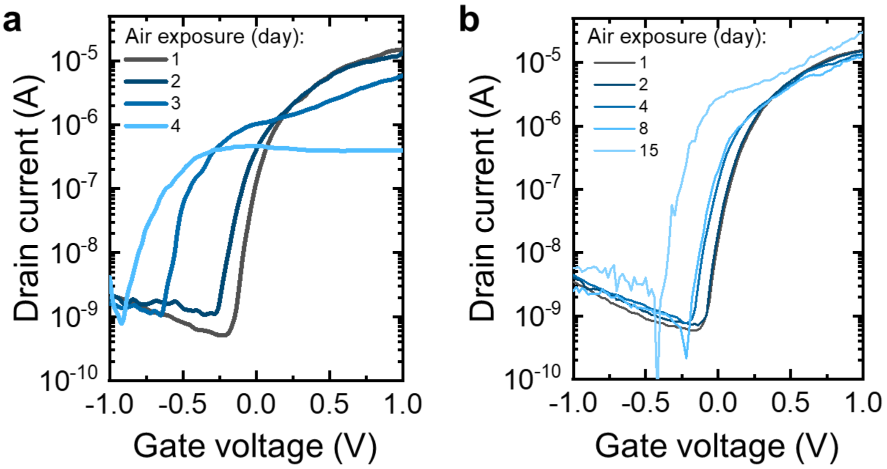

Supplementary Fig. 8. Air stability measurements
(a) unencapsulated and (b) polydimethylsiloxane-encapsulated JFETs. The devices were stored in the dark at ≈20 °C and ≈40% relative humidity.

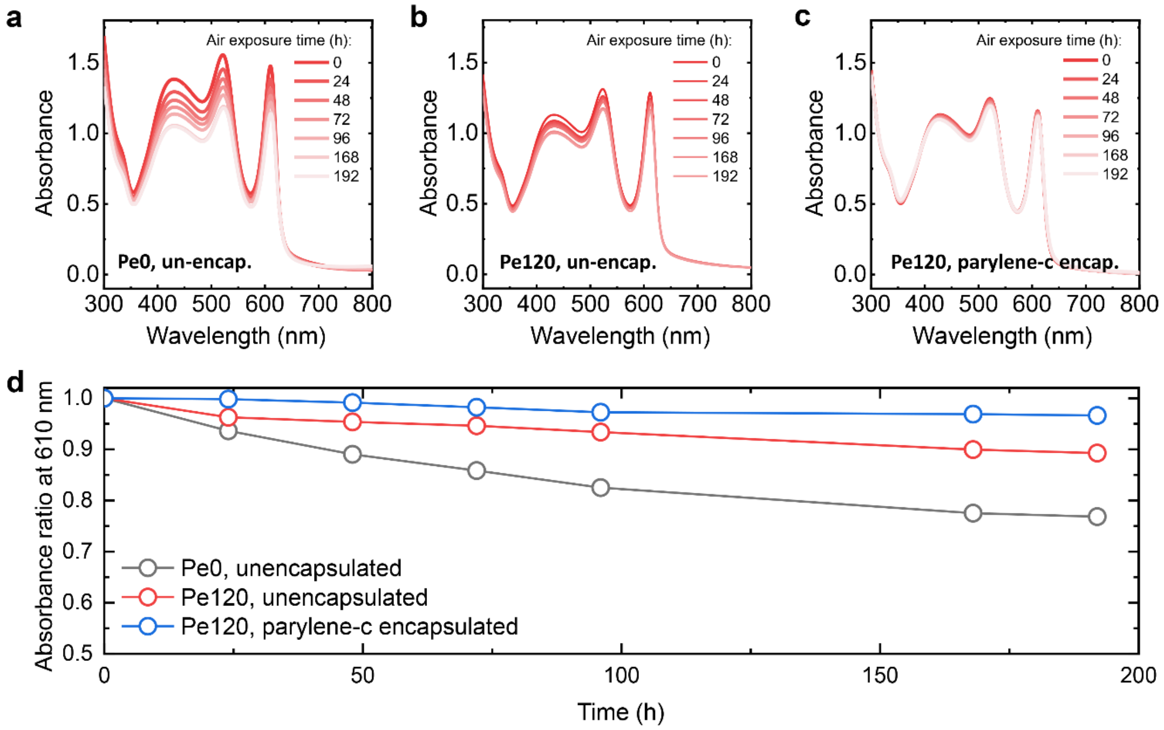

Supplementary Fig. 9. UV–visible spectra of the PEA_2SnI_4 films with different air exposure times
(a) Pristine PEA_2SnI_4 (Pe0) without encapsulation, (b) PEA_2SnI_4 annealed in the air for 120 min (Pe120) without encapsulation, and (c) Pe120 with parylene-c encapsulation. (d) Absorbance ratio at 610 nm from (a), (b), and (c) as a function of time.

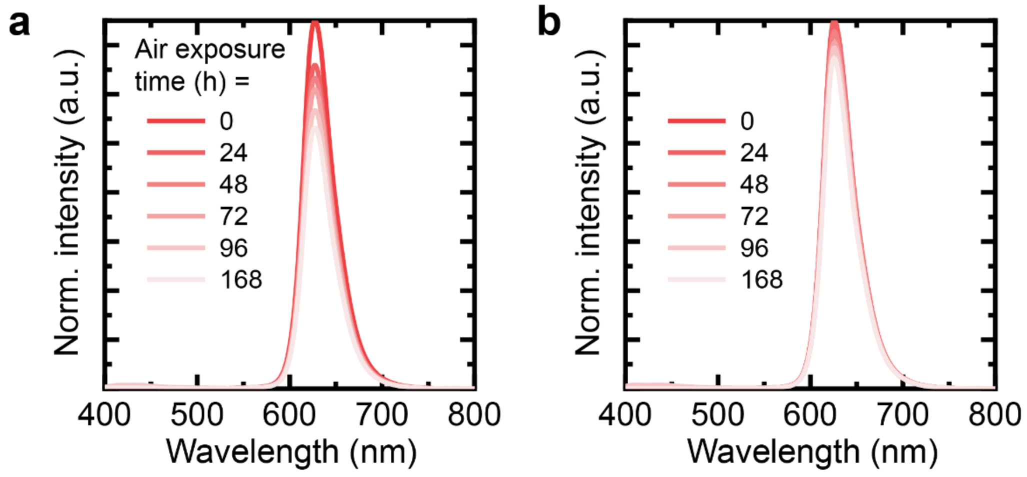

Supplementary Fig. 10. Time-dependent photoluminescence measurements of perovskite films. Time-dependent photoluminescence measurements showing degradation kinetics of (a) Pe0 and (b) Pe120 with parylene-c encapsulation.

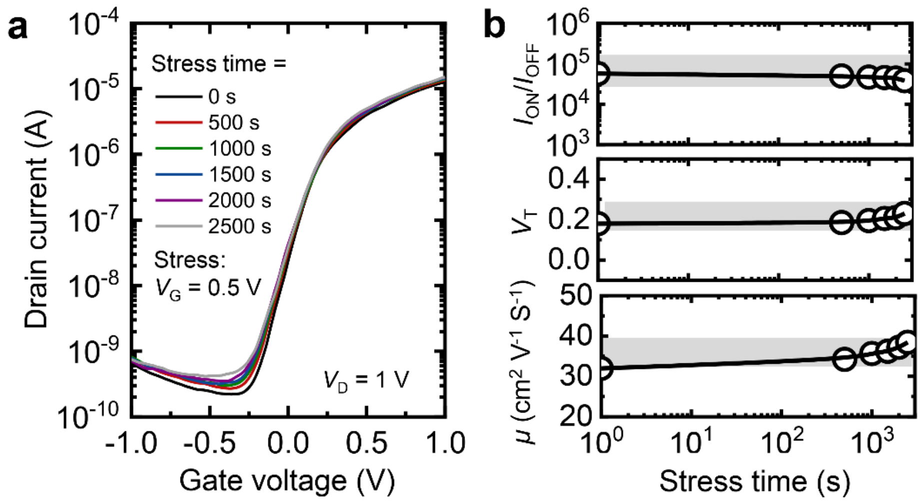

Supplementary Fig. 11. Gate bias stress stability results of the perovskite b-JFET device
(a) Gate bias stress stability results of the perovskite b-JFET device. V_{GS} sweeps from −1 V to 1 V were conducted every 500 s, during which a gate bias of 0.5 V was applied. (b) On/off ratio, threshold voltage, and effective field-effect mobility calculated during the test.

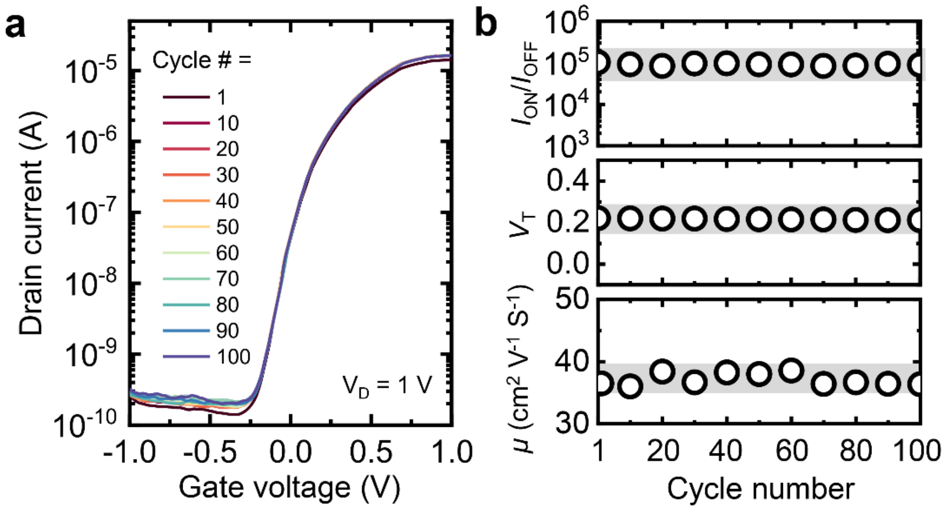

Supplementary Fig. 12. Cyclic stability results of the perovskite b-JFET device
(a) Cyclic stability results of the perovskite b-JFET device. A total of 100 cycles of V_{GS} sweeps from −1 V to 1 V was conducted. (b) On/off ratio, threshold voltage, and effective field-effect mobility calculated during the test.

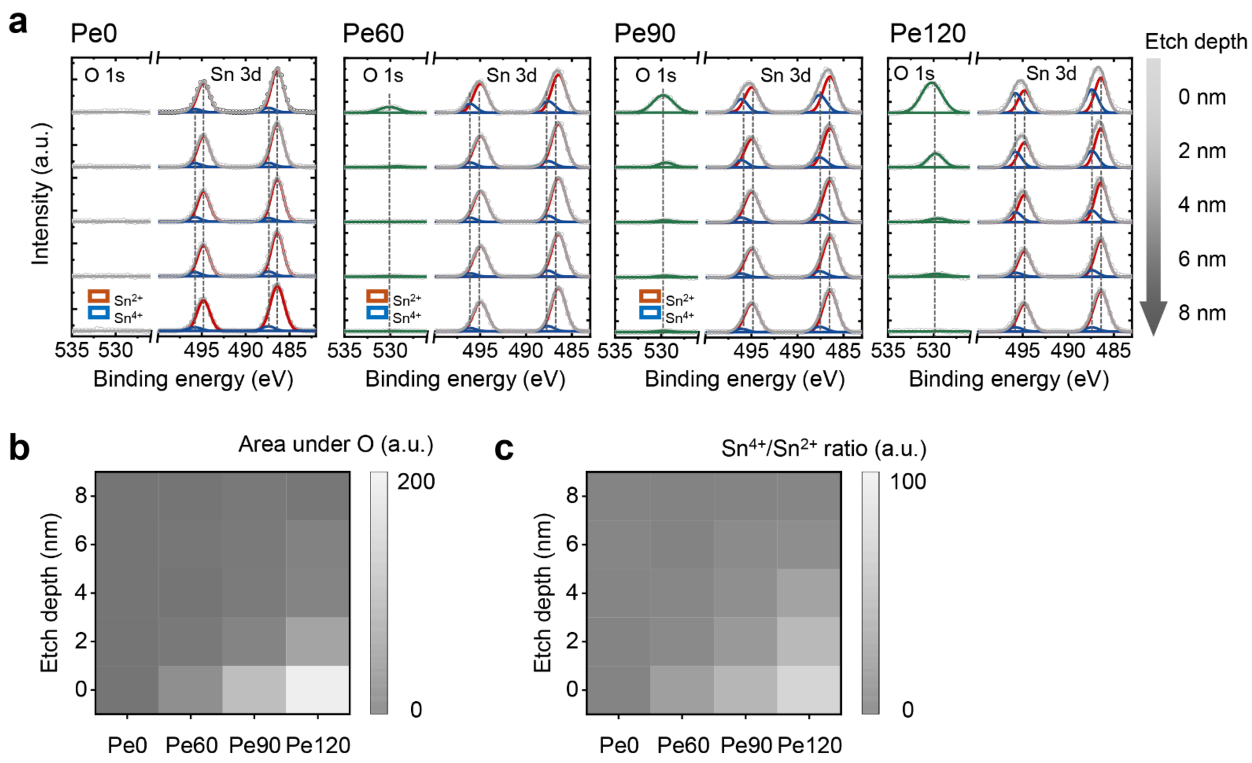

Supplementary Fig. 13. XPS depth profiles of perovskite films
(a) XPS depth profiles of Pe0, Pe60, Pe90, and Pe120 films. (b) Heat map showing oxygen area of Pe0, Pe60, Pe90, and Pe120 as a function of the etch depth. (c) Depth-dependent Sn^{4+}-to-Sn^{2+} ratios for Pe0, Pe60, Pe90 and Pe120 calculated from the deconvoluted peaks of the Sn 3d XPS spectra.

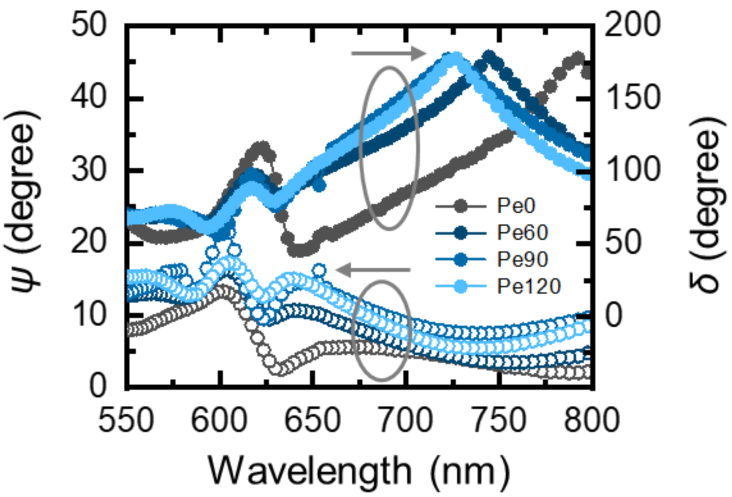

Supplementary Fig. 14. Ellipsometry measurements of perovskite films
Ellipsometry measurements showing angles of ψ and δ of Pe0, Pe60, Pe90 and Pe120 as a function of wavelength in a range of 550-820 nm.

The ellipsometry data was fitted using the Forouhi-Bloomer model to determine the thickness of both the bulk perovskite and surface oxidation layers[9,10]. The analysis employed the following equations:

$$n(E) = n_\infty + \sum_{k=1}^{N} \frac{B_{0K}E + C_{0k}}{E^2 - B_k E + C_k} \;, \quad \cdots\cdots (1)$$

$$k(E) = \sum_{k=1}^{N} \frac{A_k (E - E_g)^2}{E^2 - B_k E + C_k}, \quad \cdots\cdots (2)$$

where n and k are real and imaginary components of the refractive index, respectively, both being functions of photon energy E. E_g is the bandgap, A_i, B_i, C_i are constants related to i) the square of the position matrix element (electron transition lifetime), ii) twice the bandwidth difference between the conduction band and the valence band, iii) dependent variable based on A_i and B_i, respectively. The fitting parameters for PEA_2SnI_4 films are detailed in **Supplementary Table 1**.

The analysis revealed progressive growth of the oxidation layer: 0 nm (Pe0), 8.5 nm (Pe60), 11.7 nm (Pe90), and 16.8 nm (Pe120). In contrast, the bulk PEA_2SnI_4 layer thickness showed a decreasing trend: 163.1 nm (Pe0), 155.1 nm (Pe60), 138.3 nm (Pe90), and 130.5 nm (Pe120).

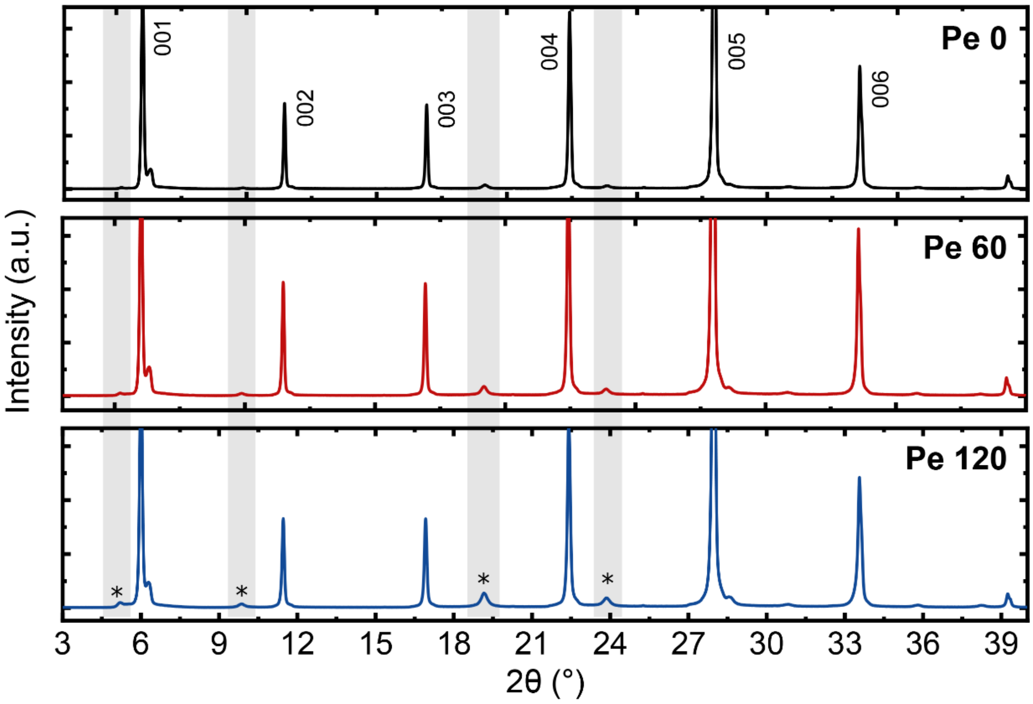

Supplementary Fig. 15. X-ray diffraction spectra of perovskite films

X-ray diffraction spectra of the Pe0, Pe60, and Pe120 thin films. The peaks corresponding to PEAI are marked with an asterisk (*) and shaded in gray.

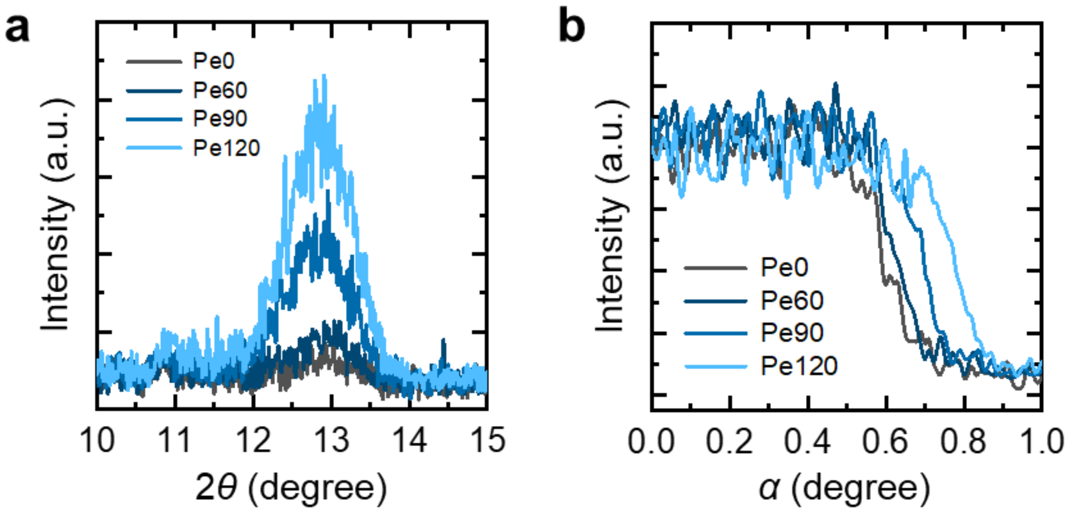

Supplementary Fig. 16. GIXRD results of perovskite films
GIXRD results of Pe0, Pe60, Pe90 and Pe120 films. (a) GIXRD data showing the evolution of the peak at $2\theta = 12.88°$ across Pe0, Pe60, Pe90, Pe120 samples, measured at a grazing incidence angle (α) of 0.7°. (b) Peak intensity at $2\theta = 12.88°$ as a function of incident angle α.

The GIXRD measurements (**Supplementary Fig. 16a**) revealed a distinctive peak at $2\theta = 12.88°$ when using a grazing incidence angle (α) of 0.7°, which was not detected in conventional XRD measurements perhaps due to the relatively low crystallinity of the component compared to the highly crystalline bulk PEA_2SnI_4 (**Supplementary Fig. 15**). The intensity of the peak showed a progressive increase from Pe0 to Pe120. We attribute this unobserved peak to SnI_2[11,12], which is presumably an intermediate product in the sequential oxidation reaction pathway of PEA_2SnI_4[13]. Furthermore, we conducted an analysis of peak intensity at $2\theta = 12.88°$ across incident angles ranging from 0° to 1° (**Supplementary Fig. 16b)**. Substantial peak intensity was monitored at higher α values for films oxidized for longer period, indicating that the surface layer yielding SnI_2 diffraction progressively grows with increased oxidation time. These results altogether provide strong evidence for the progressive growth of the surface layer with increasing oxidation time.

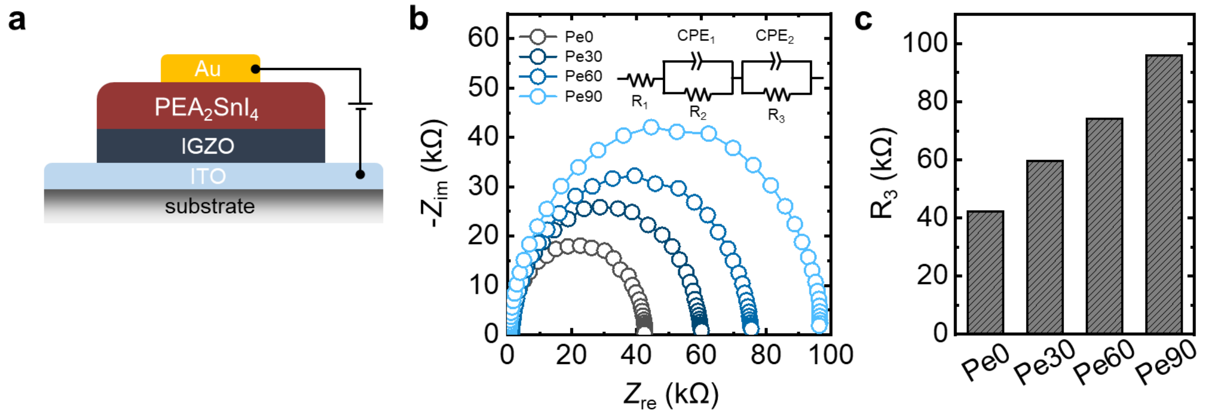

Supplementary Fig. 17. EIS measurements of the perovskite b-JFETs
(a) Device structure. (b) Nyquist plot of the EIS measurements of the Pe0-, Pe30-, Pe60-, and Pe90-based devices. The corresponding equivalent circuit is displayed in the inset. (c) Summarized R_3 values of the fitted curve.

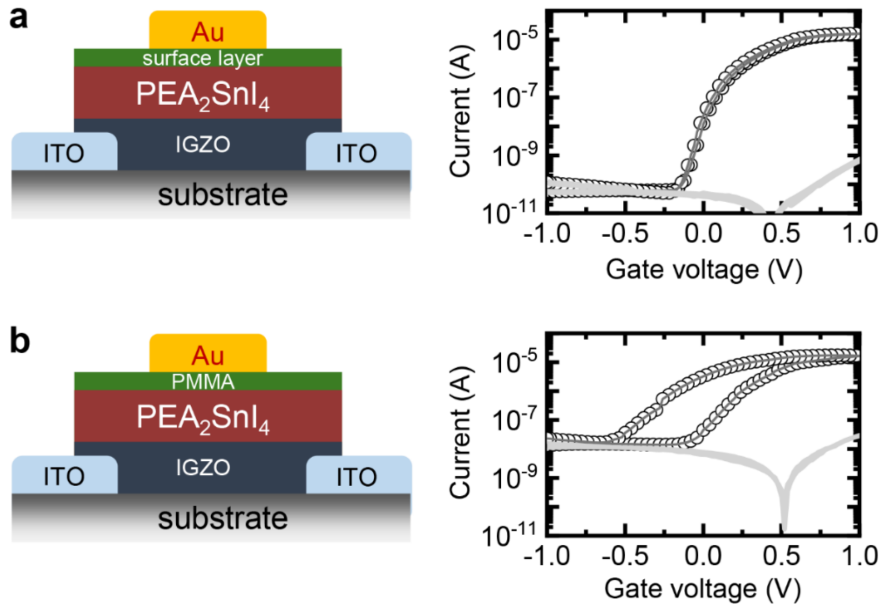

Supplementary Fig. 18. **Transfer curve comparison with PMMA top gate dielectric**
(a) Device structure of Pe120-based perovskite b-JFET (left panel) and corresponding transfer characteristics (right panel). (b) Device structure of PMMA incorporated Pe0-based perovskite b-JFET (left panel) and corresponding transfer characteristics (right panel).

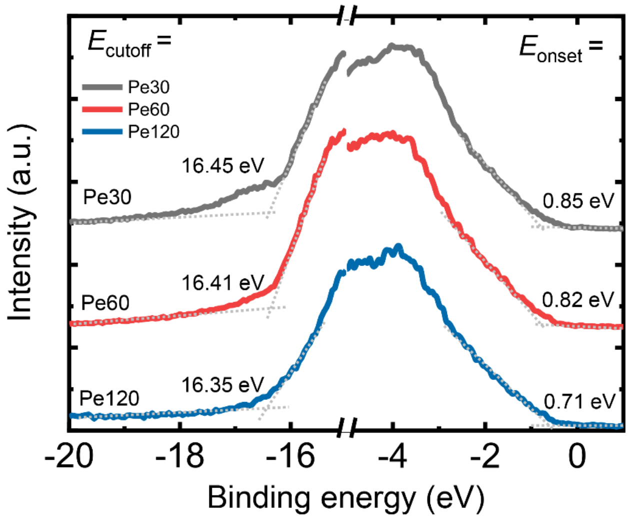

Supplementary Fig. 19. Ultraviolet photoelectron spectra of the bulk PEA_2SnI_4 films after Ar sputtering for the etching of the surface layer.

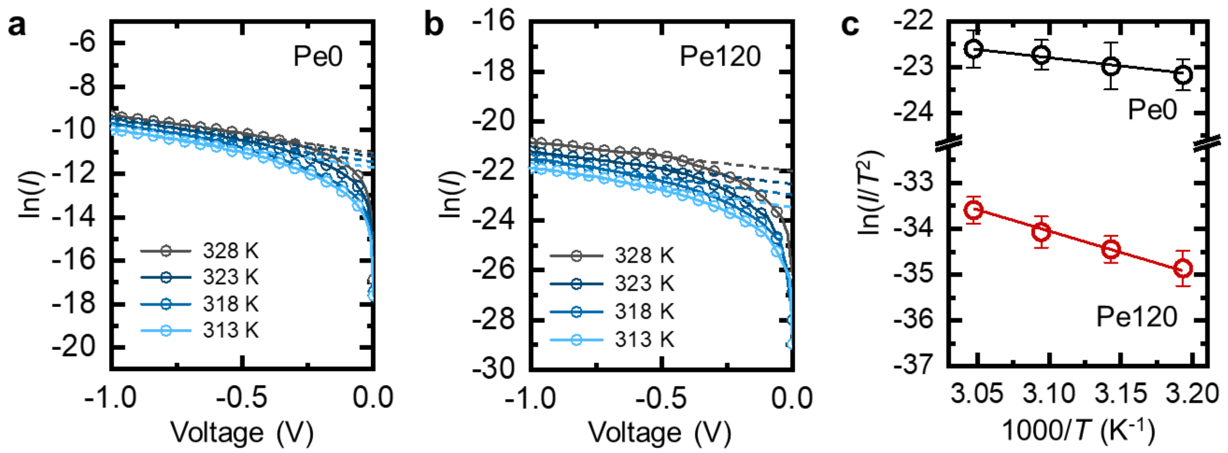

Supplementary Fig. 20. Determination of Schottky barrier heights at Au-Pe0 and Au-Pe120 junctions through thermionic emission analysis

Current-voltage (*I*-*V*) characteristics of the (a) Au-Pe0 and (b) Au-Pe120 Schottky junctions under reverse bias conditions. (c) Plots of $\ln(I/T^2)$ *versus* $1000/T$ of the Schottky diodes

The Schottky barrier heights (SBH) at the Au-Pe0 or Au-Pe120 junctions were investigated using thermionic emission theory[14]. According to this theory, the SBH (φ_{SB}) is directly related to the saturation current (I_{sat}) of a Schottky diode through the following equation:

$$I_{sat} = AA^*T^2\exp(q\varphi_{SB}/k_BT) \quad \cdots\cdots(3)$$

where A is the area of the Schottky junction, A^* is the effective Richardson constant, q is the elementary charge, k_B is Boltzmann constant, and T is the absolute temperature. I_{sat} value was determined from the y-intercept of the extrapolated lines under reverse bias conditions ($V < 0$), as shown in **Supplementary Figs. 20a** and **b**). Subsequently, φ_{SB} of for each Schottky diode was extracted from the slope of the $\ln(I_{sat}/T^2)$ *versus* $1/k_BT$ plot (**Supplementary Fig. 20c**). Analysis revealed SBH values of 0.20 eV and 0.91 eV for the Pe0-based and Pe120-based Schottky diodes, respectively. These results showed align with the UPS measurements, validating our theoretical approach.

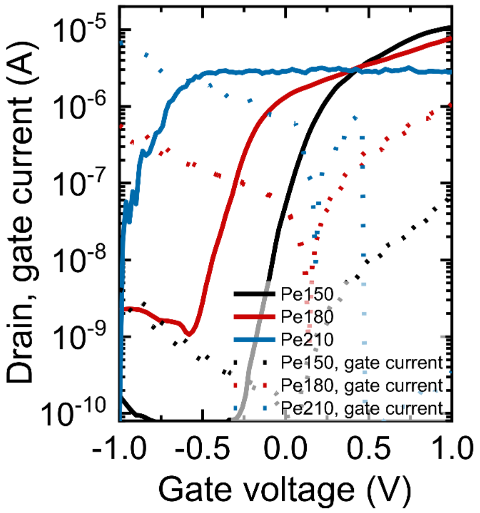

Supplementary Fig. 21. Transfer curves of the perovskite b-JFETs
Transfer curves of the (a) Pe150-, (b) Pe180-, and (c) Pe210-based perovskite b-JFETs.

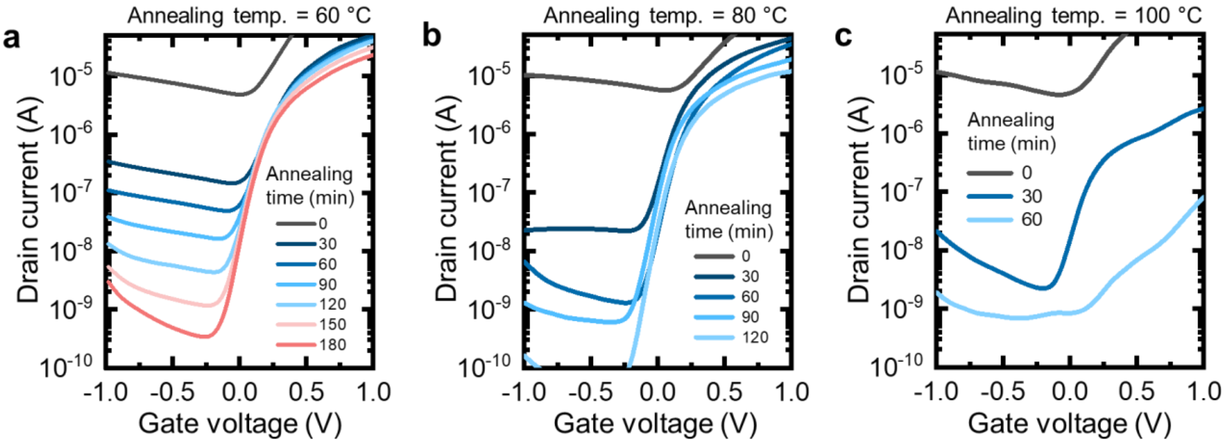

Supplementary Fig. 22. Transfer characteristics of perovskite b-JFETs as a function of annealing temperature
The annealing temperatures are (a) 60°C, (b) 80°C, and (c) 100°C.

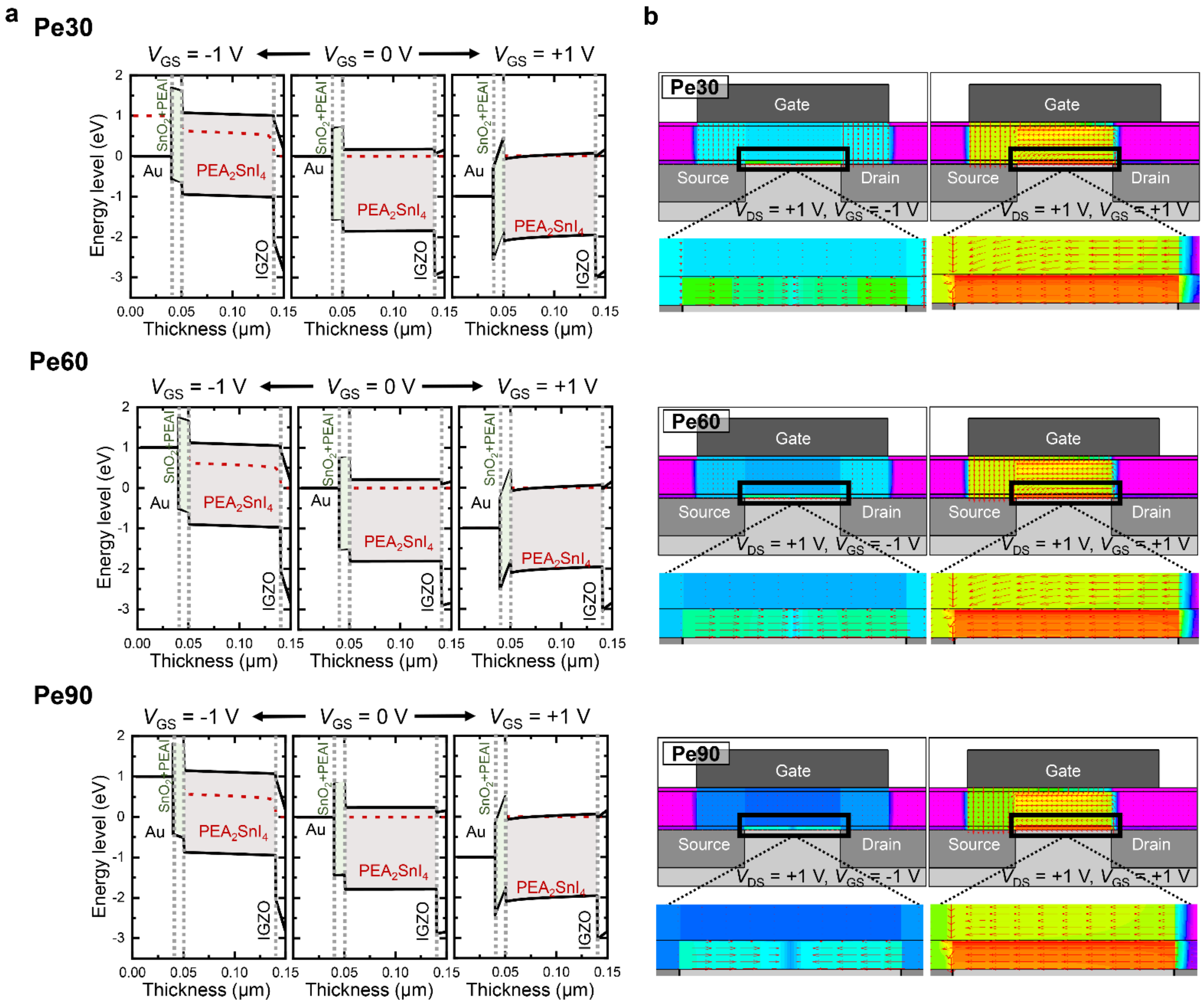

Supplementary Fig. 23. Energy band diagrams and contour maps

(a) Energy band diagrams for Pe30, Pe60, and Pe90. (b) Contour maps for the current densities of Pe30, Pe60, and Pe90.

Supplementary Table 1. Fitting parameters for ellipsometry measurements

Model parameter	PEA_2SnI_4
n_∞	2.069
E_g	1.934
A_1	0.172
B_1	4.044
C_1	4.090
A_2	0.135
B_2	10.624
C_2	27.340

Supplementary Table 2. **Benchmark comparison of IGZO FET-based inverters with solution-processed gate dielectrics**

Gate dielectric	Deposition method	Inverter gain	V_{DD}	Gain at V_{DD} = 1 V	Process temp. (°C)	Pull-up device	SS (mV/dec)	Ref
$HfGdO_x$	Spin-coating	19.8	5	3.9	450	resistor	70	[1]
PVP-co-PMMA/Al_2O_3	Spin-coating	17.3	3	5.7*	300	PMOS	-	[2]
ZrO_2	Spin-coating	10.8	5	2.2*	200	NMOS	300	[3]
ZAO	Spray-coating	58	6	9.7*	420	NMOS	115	[4]
HfAlO	Spin-coating	4.46	4	0.9	450	NMOS	87	[5]
Al_2O_3	Spin-coating	32.2	5	6.4*	-	NMOS	-	[6]
HfLaOx	Spin-coating	7.59	2.5	3.0*	300	resistor	140	[7]
**Perovskite****	**Spin-coating**	**27.4**	**1**	**27.4**	**100**	**NMOS**	**67.1**	**This work**

* Estimated value derived by normalizing the reported inverter gain to a V_{DD} of 1 V.

** Not a gate dielectric, but a p-type gate semiconductor in JFET.

Supplementary References

1 Zhang, Y., *et al.* Aqueous-solution-driven $HfGdO_x$ gate dielectrics for low-voltage-operated α-InGaZnO transistors and inverter circuits. *J. Mater. Sci. Technol.* **50**, 1-12 (2020).

2 Cho, H. J., *et al.* Solution-processed organic-inorganic hybrid gate insulator for complementary thin film transistor logic circuits. *Th. Sol. Films* **673**, 14-18 (2019).

3 Park, S.-J., Ha, T.-J. Sol-gel-based metal-oxide thin-film transistors for high-performance flexible NMOS inverters. *J. of Alloys Compd.* **912**, 165228 (2022).

4 Islam, M. M., *et al.* Spray-pyrolyzed high-k zirconium-aluminum-oxide dielectric for high performance metal-oxide thin-film transistors for low power displays. *Adv. Mater. Interfaces* **8**, 2100600 (2021).

5 He, G., Li, W., Sun, Z., Zhang, M., Chen, X. Potential solution-induced HfAlO dielectrics and their applications in low-voltage-operating transistors and high-gain inverters. *RSC Adv.* **8**, 36584-36595 (2018).

6 Park, S. J., Ha, T. J. Microwave-irradiated metal-oxide thin-film transistors with recessed gate structure and their applications in logic circuits. *IEEE Trans. Electron Dev.* **70**, 99-104 (2023).

7 Wang, W., He, G., Wang, L., Xu, X., Zhang, Y. Solution-driven $HfLaO_x$-based gate dielectrics for thin film transistors and unipolar inverters. *IEEE Trans. Electron Dev.* **68**, 4437-4443 (2021).

8 Abdulrahim, S. M., Ahmad, Z., Bahadra, J. & Al-Thani, N. J. Electrochemical impedance spectroscopy analysis of hole transporting material free mesoporous and planar perovskite solar cells. *Nanomater.* **10**, 1635 (2020).

9 Cai, S. *et al.* Fast-response oxygen optical fiber sensor based on PEA_2SnI_4 perovskite with extremely low limit of detection. *Adv. Sci.* **9**, 2104708 (2022).

10 Forouhi, A. R. & Bloomer, I. Optical dispersion relations for amorphous semiconductors and amorphous dielectrics. *Phys. Rev. B* **34**, 7018-7026 (1986).

11 Chen, Y. -S. *et al.*, Intermediate-controlled synthesis of quasi-2D $(PEA)_2MA_4Pb_5I_{16}$ in the 20–30% relative humidity glovebox environment for fabricating perovskite solar cells with 1 month durability in the air. *ACS Omega*, **9**, 48374-48389 (2024).

12 Sembito, A. *et al.*, Characterization of 2D-PEA_2SnI_4 perovskite thin films grown by sequential physical vapor deposition. *Vacuum*, **233**, 113954 (2025).

13 Ju, Y. *et al.*, The evolution of photoluminescence properties of PEA_2SnI_4 upon oxygen exposure: insight into concentration effects. Adv. Funct. Mater., **32**, 2108296 (2021).

14 Choi. Y. J. *et al.* Remote gating of schottky barrier for transistors and their vertical integration. *ACS Nano.* **13**, 7, 7877-7885 (2019).
